# *Saccharomyces cerevisiae* cellular engineering for the production of FAME biodiesel

**DOI:** 10.1186/s13568-024-01702-7

**Published:** 2024-04-24

**Authors:** Laiyou Wang, Bingbing Liu, Qingshan Meng, Chunchun Yang, Yiyi Hu, Chunyan Wang, Pengyu Wu, Chen Ruan, Wenhuan Li, Shuang Cheng, Shuxian Guo

**Affiliations:** 1 Henan Key Laboratory of Industrial Microbial Resources and Fermentation Technology, Nanyang Institute of Technology, 473004 Nanyang, China; 2 State Key Laboratory of Microbial Metabolism, Joint International Research Laboratory of Metabolic and Developmental Sciences, School of Life Sciences and Biotechnology, Shanghai Jiao Tong University, Shanghai, China

**Keywords:** Biodiesel, Fatty acid methyl esters, *Saccharomyces cerevisiae*, Free fatty acids, S-adenosylmethionine

## Abstract

The unsustainable and widespread utilization of fossil fuels continues to drive the rapid depletion of global supplies. Biodiesel has emerged as one of the most promising alternatives to conventional diesel, leading to growing research interest in its production. Microbes can facilitate the de novo synthesis of a type of biodiesel in the form of fatty acid methyl esters (FAMEs). In this study, *Saccharomyces cerevisiae* metabolic activity was engineered to facilitate enhanced FAME production. Initially, free fatty acid concentrations were increased by deleting two acetyl-CoA synthetase genes (*FAA1, FAA4*) and an acyl-CoA oxidase gene (*POX1*). Intracellular S-adenosylmethionine (SAM) levels were then enhanced via the deletion of an adenosine kinase gene (*ADO1*) and the overexpression of a SAM synthetase gene (*SAM2*). Lastly, the *S. cerevisiae* strain overproducing free fatty acids and SAM were manipulated to express a plasmid encoding the *Drosophila melanogaster* Juvenile Hormone Acid *O*-Methyltransferase (*Dm*JHAMT). Using this combination of engineering approaches, a FAME concentration of 5.79 ± 0.56 mg/L was achieved using these cells in the context of shaking flask fermentation. To the best of our knowledge, this is the first detailed study of FAME production in *S. cerevisiae*. These results will provide a valuable basis for future efforts to engineer *S. cerevisiae* strains for highly efficient production of biodiesel.

**Supplementary Information:**

The online version contains supplementary material available at 10.1186/s13568-024-01702-7.

## Key points

De novo synthesis of FAMEs was demonstrated in *Saccharomyces cerevisiae.*

FAME production was improved by increasing concentrations of fatty acids and SAM.

FAMEs produced in *S. cerevisiae* mainly included C16 and C18 fatty acid methyl esters.

**Supplementary Information:**

The online version contains supplementary material available at 10.1186/s13568-024-01702-7.

## Introduction

Ongoing concerns about the impact of global climate change and energy security have widespread implications for environmental integrity, sustainability, and food security (Kaljuvee and Kuusik 1999; Kotcher et al. 2019). In an effort to address fossil fuel depletion, pollution, and the negative effects of these fuels on the climate, there is an urgent need to move away from fossil fuel dependency in favour of renewable energy sources (Khan and Fu 2020). Biodiesel has emerged as a promising fossil fuel alternative with the potential to be produced in an ecologically friendly manner (Alishah Aratboni et al. 2019; Snowdon R Fau - Friedt 2012).

Biodiesel consists of the ethyl- and/or methyl-esters of linear alkyl chains, and serves as a clean-burning renewable fuel that can be directly used in current diesel engines without any need for additional modifications (Nady et al. 2020). As compared to fossil fuels, biodiesel exhibits excellent ignition properties and can reduce the emission of CO_2_ and other fumes by 78% (Elkelawy et al. 2022).

Biodiesel production is primarily achieved via the transesterification of animal fat- or vegetable oil-derived triacylglyceride oils with methanol or ethanol using an alkaline catalyst (Leung et al. 2010). However, as these oils are also consumed by humans, using them to produce biodiesel would result in higher prices for both vegetable oil and the resultant biodiesel (Verma and Kuila 2020). The crops required to produce these oils also require extensive agricultural space and an extended growth period (Zhang et al. 2021). To overcome these issues, research groups have explored the production of biodiesel using yeast, fungi, and algae capable of lipid biogenesis (Bhatia et al. 2017; Rasmey et al. 2020; Zhang et al. 2021). The microbe-based production of biodiesel necessitates access to large-scale cultivation and harvesting systems (Rasmey et al. 2020). The growth of these microbes needs to be regulated with great care to establish optimal conditions for large-scale biodiesel generation. The establishment of metabolically engineered microbial species capable of directly synthesizing biodiesel represents a promising alternative to the use of biodiesel derived from plant or animal fats.

The Steinbüchel group made the first effort to achieve fatty acid ethyl esters (FAEEs) synthesis in *Escherichia coli* (Elbahloul and Steinbüchel 2010). To achieve this goal, *E. coli* was engineered to use two orthogonal pathways to synthesize fatty acyl-CoA and ethanol, with these substrates then processed by a wax ester synthase to facilitate the synthesis of FAEEs (Elbahloul and Steinbüchel 2010). The Keasling group further optimized *E. coli-*mediated FAEEs synthesis, achieving biodiesel yields of 1.5 g/L (Zhang et al. 2012). To simplify biodiesel synthesis pathways, a *Mycobacterium marinum* (MmMT)-derived S*-*adenosyl-*L-*methionine (SAM)-dependent methyltransferase was introduced into *E. coli* by the Lykidis group to directly catalyze endogenous fatty acid and SAM reactions to facilitate fatty acid methyl esters (FAMEs) production (Nawabi et al. 2011). However, the MmMT methyltransferase exhibits a high degree of specificity with a preference for less common fatty acids harboring a 3-hydroxy group, resulting in low FAMEs yields (Nawabi et al. 2011). To address this issue, the Saken group identified *Drosophila melanogaster* Juvenile Hormone Acid O-Methyltransferase (*Dm*JHAMT) as an enzyme with broader fatty acid specificity (Sherkhanov et al. 2016). The introduction of *Dm*JHAMT into *E. coli* and the further enhancement of intracellular SAM supply levels was sufficient to increase FAME yields to 0.56 g/L (Sherkhanov et al. 2016).

*Saccharomyces cerevisiae* has been extensively studied as a model microorganism and has many advantageous characteristics that make it well suited to commercial use, including resistance to phage contamination, the ability to undergo high-density fermentation, and a high level of robustness suitable for growth under industrial conditions (Jin and Cate 2017; Leber et al. 2015; Lian and Zhao 2015). These characteristics have led to growing interest in its use as a host to facilitate fatty acid-derived chemical and biofuel production. Under normal conditions, C16 and C18 fatty acids comprise the majority of the lipid content present within *S. cerevisiae* cells, and these fatty acids are suitable precursors that can be used to generate biodiesel (Keasling 2013; Leber et al. 2015). Accordingly, several research teams have engineered *S. cerevisiae* to produce FAEEs via the heterologous expression of wax ester synthase or an acyl-CoA: alcohol acyltransferase (De Jong et al. 2015; Valle-Rodríguez et al. 2014; Zhou et al. 2016). To date, however, there have been no reports of the metabolic engineering of yeast to facilitate FAMEs production.

In the present study, *S. cerevisiae* cells were engineered to achieve FAMEs production. Initially, genetic engineering was used to modulate the free fatty acid and SAM metabolic pathways to increase the levels of these FAME precursors. The *Dm*JHAMT methyltransferase was then introduced into the resultant free fatty acid- and SAM-overproducing *S. cerevisiae* strain. Together, this combined engineering strategy was used to successfully achieve the first reported instance of de novo FAMEs synthesis in *S. cerevisiae.*

## Materials and methods

### Strains and growth conditions

All strains used in this study were detailed in Table S1. *E. coli* DH5α strains were cultured at 37 °C and 200 rpm using LB complex medium (10 g/L tryptone, 5 g/L yeast extract, and 5 g/L NaCl) containing ampicillin (100 mg/L) when appropriate. *S. cerevisiae* was cultured in YPD complex medium (10 g/L yeast extract, 20 g/L peptone, and 20 g/L glucose) at 30 °C before transformation. After transformation, yeast cells were grown in appropriate synthetic complete (SC) medium minus the auxotrophic compound (FunGenome Company, Beijing, China) complemented by the plasmids. To prepare solid LB or YPD media, agar (20 g/L) was included.

### Primers and plasmid construction

All plasmids used for the present study were detailed in Table S1, while all primers were presented in Table S2. All plasmid constructions were achieved with DNA assembler. To construct the gRNA delivery vector, the *S. cerevisiae* SNR52p promoter, the synthesized crRNA, 20-bp complementary region (N20) and SUP4t were assembled into the *Not*I/*EcoR*I site of pDB78 to generate pDB78-X (a generic term of all gRNA delivery vector and “X” represents the serial number). The approach was used to produce the pDB78-1 (pDB78-SNR52p-*FAA*1gRNA), pDB78-2 (pDB78-SNR52p-*FAA*4gRNA), pDB78-3 (pDB78-SNR52p-*POX*1gRNA), pDB78-4 (pDB78-SNR52p-*SAM2*gRNA), and pDB78-5 (pDB78-SNR52p-*ADO1*gRNA) vectors.

To construct the *Dm*JHAMT methyltransferase-encoding vector, *S. cerevisiae* genomic DNA was used to amplify the TPI1p promoter, while the codon-optimized *DmJHAMT* gene fragments were synthesized by GenScript (Nanjing, China). The synthesized terminator named Tguo1, which is only 39 bp long and enables an ease of cloning using inexpensive oligos, was introduced into the primer P48 for amplifying the gene *DmJHAMT* (Curran et al. 2015). Therefore, the amplified DNA fragment had the terminator Tguo1 at the end.

The two TPI1p and *DmJHAMT* fragments were then assembled in the pESC-HIS vector using the *Bam*HI/*EcoR*I site to produce the pESC-HIS-1 plasmid.

### Genetic manipulation

Homologous upstream and downstream arms (∼0.5 kb each) for target genes of interest were amplified from genomic DNA prepared from *S. cerevisiae* and jointed by overlap-extension PCR to be used as editing templates. Yeast transformation was performed using the lithium acetate method as described for *S. cerevisiae* (Gietz et al. 2002). A CRISPR/Cas9 approach was used for markerless genome editing. The schematic process of genome editing was shown in Fig. S1. Briefly, the Cas9 expression plasmid pCRCT was first introduced into the strain YPH499. After transformation, cells were plated on selective media (SC-uracil) and allowed to grow for 3–4 days until colonies appeared. Colony PCR was performed to verify the transformants. To obtain a knockout strain, the prepared gRNA delivery vector and editing template were introduced into Cas9 expressing cells. The resulting transformants were screened on selective media (SC-uracil and histidine). Eight to twelve colonies for each mutant were randomly selected and colony PCR was used to verify the success of genomic editing. Once the desired mutation was verified, the gRNA delivery vector containing the *HIS1* marker was eliminated by culturing the strains in SC-uracil medium. The mutant could then be used for the next round of genome editing. When all gene manipulations were complete, the Cas9 expression vector containing the *URA3* marker was eliminated by culturing the strains in YPD medium without selection pressure.

### Flask fermentation

Individual colonies were used to inoculate 10 mL of SC or YPD media, followed by culture for ∼24 h at 30℃. After the preculture stage was complete, seed cultures were used to inoculate 100 mL of SC or YPD media in a 500 mL Erlenmeyer flask, adjusting the OD_600_ to 0.2 following inoculation. All assays were conducted in triplicate.

### SAM assay

SAM extraction from fermentation broth was performed following the addition of 10% (w/v) perchloric acid with shaking a 220 rpm for 1 h at 30 °C. Lysates were then centrifuged (5 min, 13,000 rpm, 4 °C) to clarify the sample, after which high-performance liquid chromatography (HPLC) was used to analyze these samples with a C-18 column (Hypersil BDS 5 m 4.6 mm × 250 mm) (Chen and Tan 2018). These analyses were performed using a mobile phase consisting of 40 mmol/L NH_4_H_2_PO_4_, 2 mmol/L sodium heptyl sulfonate, and 18% methanol (Chen and Tan 2018). UV detection was performed at 254 nm. Triplicate analyses were conducted for all measurements. To measure the production of SAM, the standard curve for SAM was constructed. A series of standard solutions containing SAM (20, 40, 60, 120 and 240 mg/L) were analysed by HPLC. A least-squares fit was performed using the peak areas as the vertical coordinate and the concentration of SAM as the horizontal coordinate. SAM standard was purchased from Aladdin Ltd, Shanghai, China.

### FFAs and FAMEs extraction

Total FFAs were extracted as described by Zhang et al. (Zhang et al. 2020). Briefly, 200 µL of cell culture was mixed with 10 µL 40% tetrabutylammonium hydroxide and 200 µL methylation reagent. The methylation reagent contained 200 mM methyl iodide as methyl donor. Prior to the methylation reaction, pentadecanoic acid was added as an internal standard to the mixture of methylation reagent and sample to a final concentration of 5 mg/L. The mixture was shaken for 45 min and then centrifuged at 6000 g for 5 min. 100 µL of the dichloromethane layer was then transferred to glass GC vials for subsequent analysis. FAMEs were extracted as described by Sherkhanov et al. (Sherkhanov et al. 2016). Briefly, 5 mL of cell culture was mixed with 6 mL of a 2:1 chloroform/methanol mixture.

### FFAs and FAMEs quantification

A GC-FID approach was used to quantify levels of FFAs and FAMEs with an HP 5890 Series II gas chromatograph equipped with an HP-Innowax Column (0.32 mm × 30 m × 0.25 μm, Agilent). The injection volume for all samples was 1 µL, and other analytical parameters were as follows: inlet temperature 250 °C, split ratio 1:1, helium carrier gas at a 5 mL/min flow rate. The oven temperature was initially set to 160 °C for 3 min, after which it was increased at 5 °C/min to 255 °C and held for 3 min. The inlet temperature was 270 °C, while the detector temperature was 330 °C (Sherkhanov et al. 2016). FAME products were identified via a GC/mass spectrometry approach with an Agilent 6890 − 5975 instrument equipped with HP-Innowax Column (0.32 mm × 30 m × 0.25 μm, Agilent). Peaks were identified by comparing the results with GC retention times, known standards, and mass spectra included in the National Institute of Standards and Technology (NIST) database.

To measure the production of fatty acids and FAMEs, standard curves for the five kinds of FAMEs (C14:0, C16:0, C16:1, C18:0 and C18:1) were constructed. Briefly, a series of standard solutions containing five mixed FAME standards and internal standard methyl pentadecanoate (5 mg/L) were analysed by GC. In the standard solutions, the concentration gradients of each FAME were 1, 5, 10, 15, 20, 25 mg/L. A least-squares fit was performed using the ratio of the peak areas of the FAMEs to the peak areas of the internal standard as the vertical coordinate and the concentration of the FAME standards as the horizontal coordinate. FFA and FAME levels were analyzed by comparing the results to standard curves. FAME standards were purchased from Sigma-Aldrich.

## Results

### Improving fatty acid production

**Fig. 1:**
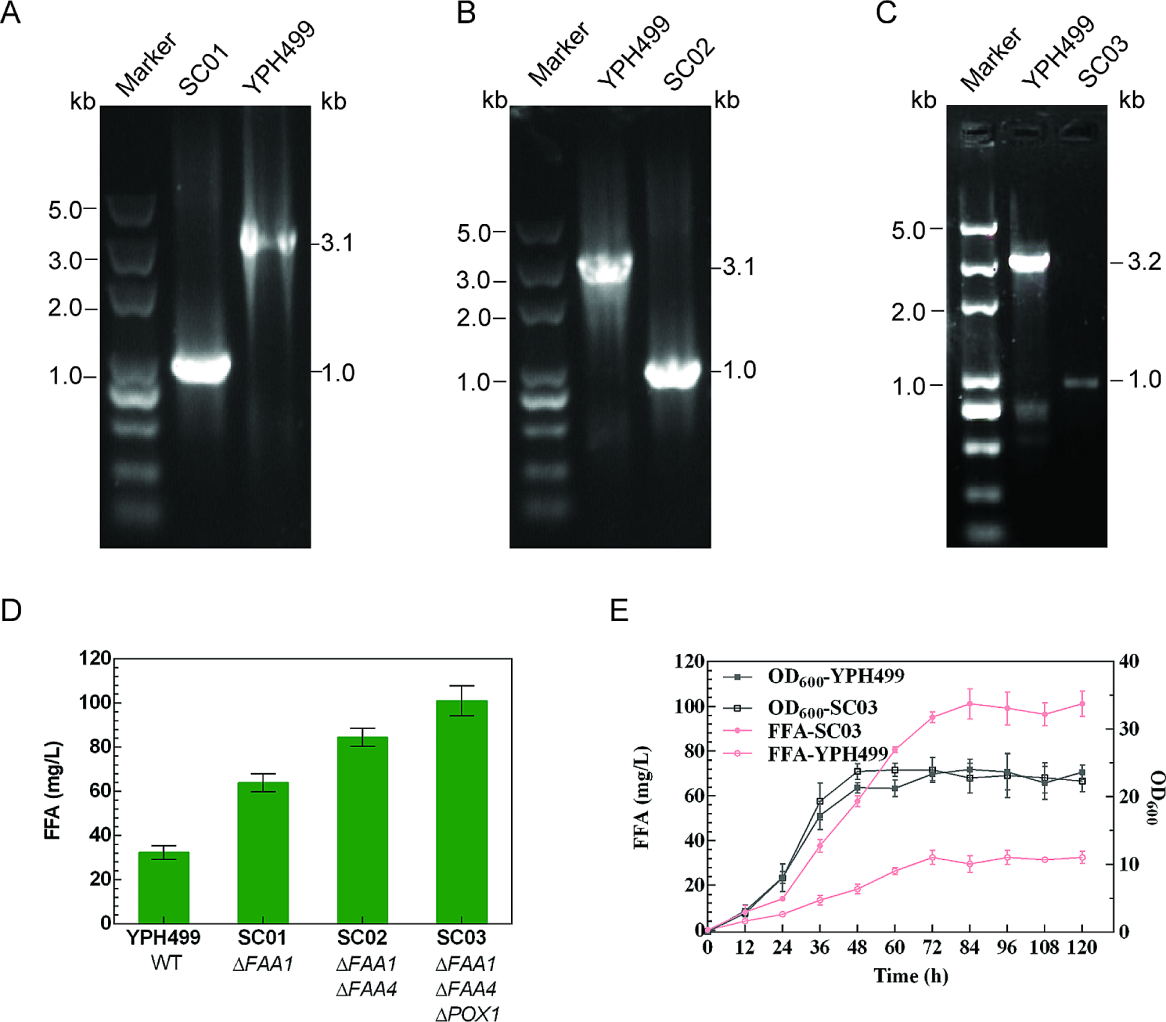
The improvement of fatty acids production. PCR identifications for the deletion of *FAA1***(A)**, *FFA4***(B)** and *POX1***(C)** genes, respectively. The Δ*FAA1* mutant yielded a 1000-bp fragment, while the YPH499 yielded a 3103-bp fragment. The Δ*FAA4* mutant yielded a 1000-bp fragment, while the YPH499 yielded a 3085-bp fragment. The Δ*POX1* mutant yielded a 1000-bp fragment, while the YPH499 yielded a 3247-bp fragment. **D**: FFA titres obtained with different engineered strains. Data are mean ± standard deviation (error bars) of three assays. **E**: Curves of cell growth and FFA productions of the mutant strain SC03 and the wild-type strain in shake flask culture

Fatty acids serve as a primary substrate for FAME synthesis, and efforts to enhance fatty acid accumulation are thus essential to facilitate the efficient production of FAMEs. Fatty acid concentrations can be reliably increased by reducing their consumption by altering metabolic pathways. In the cytosol of *S. cerevisiae* cells, the enzymes encoded by the *FAA1* and *FAA4* genes are primarily responsible for converting free fatty acids into acyl-CoA, while the *POX1* gene encodes an enzyme which initiates the process of β-oxidation via the conversion of acyl-CoA into trans-2-enoylCoA within the peroxisomal compartment (Chen et al. 2014; Zhou et al. 2016). As such, the sequential deletion of *FAA1*, *FAA4*, and *POX1* was performed in *S. cerevisiae* as a means of enhancing free fatty acid accumulation. PCR amplification was used to identify the resultant mutants (Fig. 1A-C and Fig. S2A-C), and fatty acid production in these mutants was assessed by comparison of GC analysis results with FAME standard curves (Fig. S3). As shown in Fig. 1D, the strain SC01 (Δ*FAA1*) was able to produce fatty acids at a concentration of 64.03 ± 4.03 mg/L, which was 97.32% greater than the yield from the parental strain YPH499 (32.45 ± 3.07 mg/L). The double-knockout strain SC02 (Δ*FAA1*Δ*FAA4*) exhibited a fatty acid yield of 84.58 ± 4.05 mg/L, demonstrating that *FAA4* deletion further enhanced fatty acid production. The highest fatty acid yield was observed for the triple-knockout strain SC03 (Δ*FAA1*Δ*FAA4*Δ*POX1*), which produced fatty acids at a concentration of 101.04 ± 6.81 mg/L, 2.11-fold higher than the yield of the parental strain YPH499 (Fig. 1D, E). Importantly, the deletion of the *FAA1*, *FAA4*, and *POX1* genes had no significant effect on the growth of these cells (Fig. 1E).

### Increasing SAM levels

**Fig. 2:**
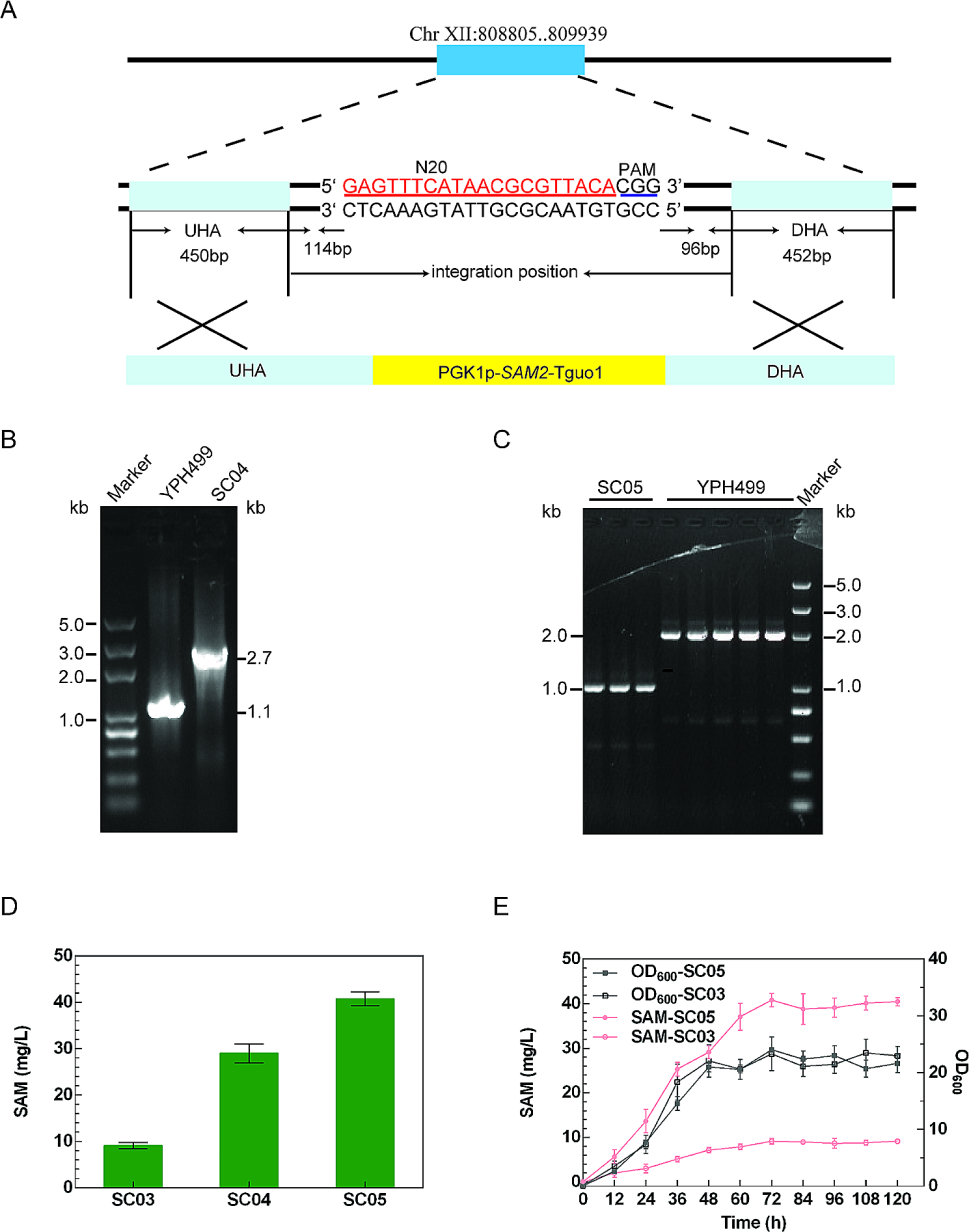
The improvement of SAM production. **(A)**: Precise integration site of *SAM2* expression cassette. PCR identifications for the integration of *SAM2* expression cassette **(B)** and the deletion of *ADO1*gene **(C)**, respectively. The integrated mutant yielded a 2712-bp fragment, while the YPH499 yielded a 1135-bp fragment. The Δ*ADO1* mutant yielded a 1000-bp fragment, while the YPH499 yielded a 2023-bp fragment. **D**: SAM titres obtained with different engineered strains. **E**: Curves of cell growth and SAM productions of the mutant strain SC05 and the strain SC03 in shake flask culture

In addition to free fatty acids, SAM is another important substrate for FAME synthesis, with intracellular SAM concentrations being a limiting factor in FAME biosynthesis. S-adenosylmethionine synthetase, encoded by the *S. cerevisiae SAM1* and *SAM2* genes, catalyzes the processing of methionine and ATP to produce SAM. SAM accumulation, however, can repress *SAM1* activity whereas the activity of *SAM2* is not responsive to SAM levels (Kanai et al. 2017). Therefore, in the present study, *SAM2* was overexpressed by introducing an extra copy into the SC03 genome to generate the integrant mutant strain SC04 (YPH499 Δ*FAA1* Δ*FAA4* Δ*POX1* XII-2::(PGK1p-*SAM2*). The chosen integration site was a region on chromosome XII (808805.909936) in an effort to minimize the effects of integration on strain fitness as a consequence of any adverse effects on nearby genes (Fig. 2A) (Mikkelsen et al. 2012). The mutant was verified via PCR (Fig. 2B and Fig. S4A). Prior studies have also reported significant increases in SAM concentrations in *S. cerevisiae* following the disruption of the *ADO1* gene, which encodes adenosine kinase (Kanai et al. 2017). Accordingly, the *ADO1* gene was deleted from the strain SC04 to yield the strain SC05 (YPH499 Δ*FAA1* Δ*FAA4* Δ*POX1* XII-2::(PGK1p-*SAM2*) Δ*ADO1*), as confirmed via PCR (Fig. 2C and Fig. S4B). Validated mutants were cultured in shaker flasks, and SAM production was assessed by comparison of HPLC analysis results with standard curves (Fig. S5 and S6). The SC04 integrant mutant was able to produce 29.03 ± 2.04 mg/L SAM (Fig. 2D), with this yield being 2.18-fold higher than that obtained from strain SC03 (9.13 ± 0.64 mg/L). *ADO1* deletion further enhanced SAM production such that the SAM yield from strain SC05 was 3.47-fold higher than that from strain SC03 (40.79 ± 1.49 mg/L) (Fig. 2D and E).

### FAME synthesis in *S. cerevisiae*

**Fig. 3:**
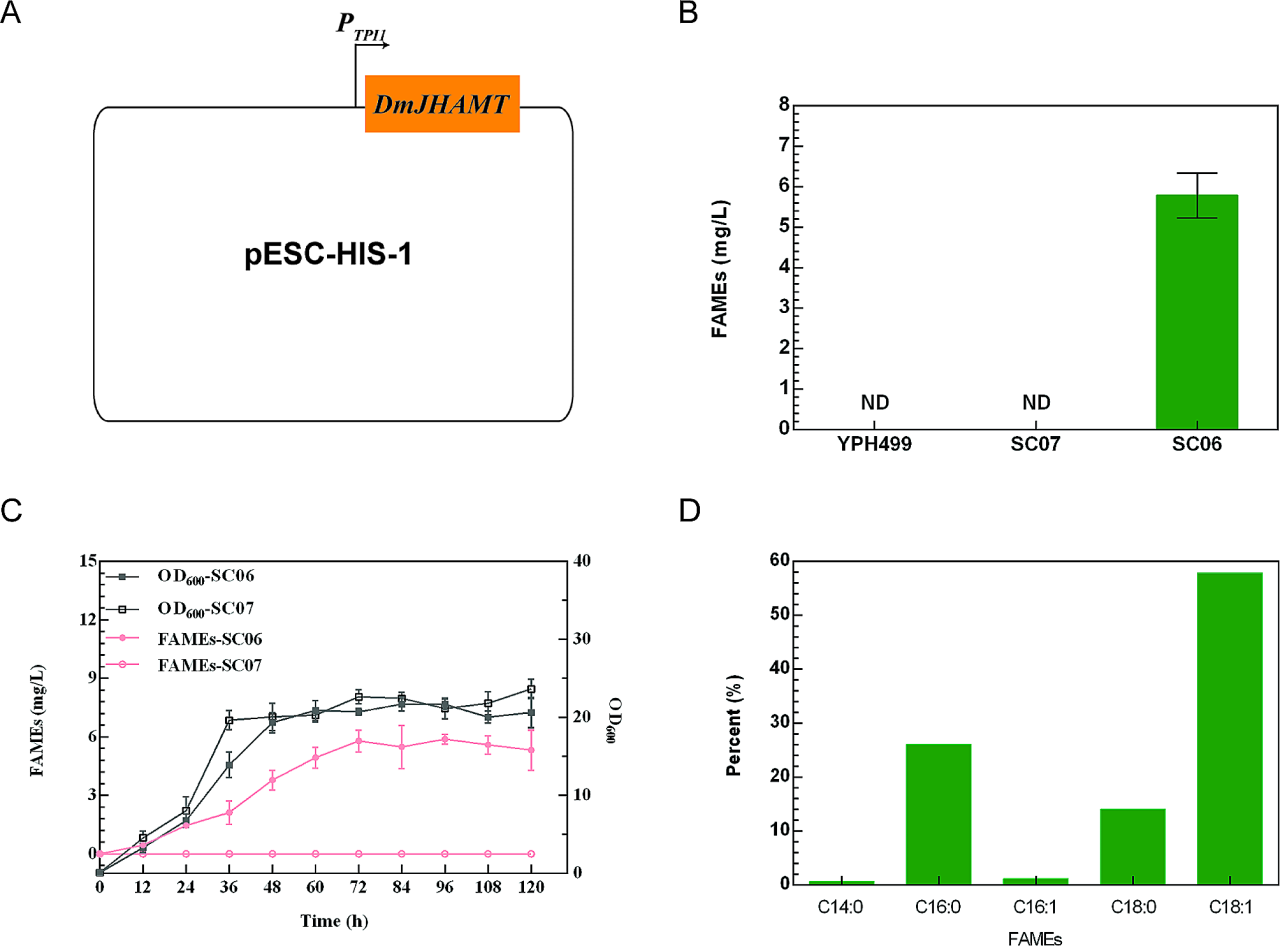
FAMEs production in *S. cerevisiae.***A**: The *Dm*JHAMT delivery vector pESC-HIS-1. The expression of *DmJHAMT* was driven by the promoter *P*_*TPI1*_. **B**: FAMEs titres obtained with different engineered strains. ND, Not Detected. **C**: Curves of cell growth and FAMEs productions of the mutant strain SC06 and the strain SC07 in shake flask culture. **D**: Profiles of FAMEs produced in SC06

The *D. melanogaster-*derived *Dm*JHAMT methyltransferase is capable of catalyzing the production of FAMEs from free fatty acids and SAM within microbial cells (Sherkhanov et al. 2016). To achieve FAME production in *S. cerevisiae*, the pESC-HIS-1 plasmid containing the *P*_*TPI1*_-controlled *DmJHAMT* gene was prepared (Fig. 3A). This plasmid was subsequently used to transform the strains SC05 and YPH499, yielding the respective strains SC06 (SC05/pESC-HIS-1) and SC07 (YPH499/pESC-HIS-1). Shaker flask cultures of strains SC06, SC07 and YPH499 were then prepared. While no FAMEs were detectable following the shaker flask cultivation of the strains SC07 or YPH499, a FAME yield of 5.79 ± 0.56 mg/L was obtained from the strain SC06 (Fig. 3B, C), in the first reported instance of FAME biosynthesis in *S. cerevisiae.* The FAMEs generated by strain SC06 included both saturated (C14:0, C16:0, C18:0) and unsaturated (C16:1 and C18:1) FAMEs, the two most abundant of which were methyl palmitate (C16:0) and methyl oleate (C18:1) (Fig. 3D).

## Discussion

Biodiesel has emerged as a promising alternative to fossil fuels and attracted increasing attention from researchers (Ramos et al. 2022; Sharma et al. 2020). Several research teams have achieved de novo microbe-mediated biodiesel production (Table 1). In *E. coli*, an FAEE yield of up to 1.5 g/L has been reported, and is the highest FAEE yield to date (Zhang et al. 2012). The highest measured FAEE yield in *S. cerevisiae* is 0.52 g/L (Yu et al. 2012). Unlike FAEEs, however, there have been far fewer studies focused on FAME synthesis. Yunus et al. engineered cyanobacteria to facilitate the conversion of CO_2_ into FAMEs at a yield of up to 120 mg/L in 10 days (Yunus et al. 2020). In *E. coli*, Nawabi et al. were able to induce FAME biosynthesis via the introduction of exogenous methyltransferases, although the utilized methyltransferase exhibits a high degree of specificity for rare 3-hydroxy group-containing fatty acids, contributing to poor FAME yields (Nawabi et al. 2011). To address this issue, Sherkhanov et al. identified *Dm*JHAMT as a broad-spectrum methyltransferase, and they introduced this methyltransferase into *E. coli* that had been engineered to overproduce SAM and fatty acids, resulting in the production of 0.56 g/L of FAMEs (Sherkhanov et al. 2016). No studies to date, however, have reported on the use of *S. cerevisiae* for FAME biosynthesis, which was achieved for the first time in the present study.

**Table 1:** Comparisons of biodiesel production from different organisms

Microorganisms	Products	Titre	Cultivation condition	Reference
*E. coli*	FAEEs	1.5 g/l	Shake flask	(Zhang et al. 2012)
*S. cerevisiae*	FAEEs	230 mg/L	Shake flask	(Teo et al. 2015)
*S. cerevisiae*	FAEEs	25 mg/L	Shake flask	(Thompson and Trinh 2014)
*S. cerevisiae*	FAEEs	6.3 mg/L	Shake flask	(Shi et al. 2012)
*S. cerevisiae*	FAEEs	5 mg/L	Shake flask	(Runguphan and Keasling 2014)
*S. cerevisiae*	FAEEs	34 mg/L	Shake flask	(Shi et al. 2014)
*S. cerevisiae*	FAEEs	17.2 mg/L	Shake flask	(Valle-Rodríguez et al. 2023)
*S. cerevisiae*	FAEEs	0.52 g/L	Shake flask	(Yu et al. 2012)
*E. coli*	FAMEs	0.56 g/L	Shake flask	(Sherkhanov et al. 2016)
*E. coli*	FAMEs	70.5 μm	Shake flask	(Nawabi et al. 2011)
*Synechocystis* sp.	FAMEs	120 mg/L	Shake flask	(Yunus et al. 2020)
*S. cerevisiae*	FAMEs	4.69 mg/L	Shake flask	This study

**Fig. 4:**
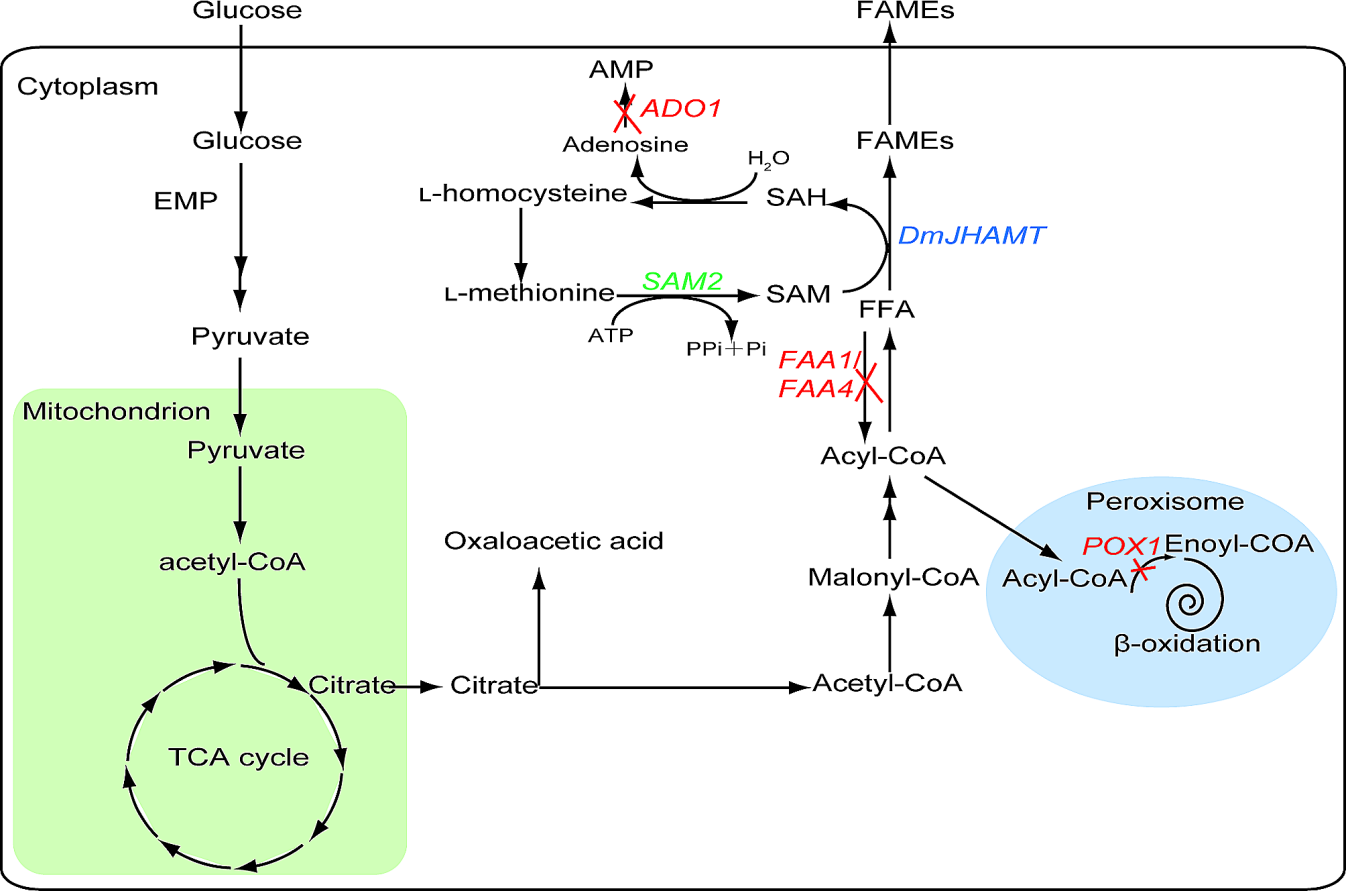
Fatty acid synthesis pathway in *S. cerevisiae* and engineering strategies for biodiesel synthesis. Overexpression targets are shown in green, and knockout targets are in red. Metabolic pathways that need to be blocked are marked with a cross. Double arrows represent multiple steps. EMP, Embden-meyerhof pathway; TCA, tricarboxylic acid; pyruvate decarboxylase; SAM, S-adenosylmethionine; SAH, S-adenosine homocysteine; FFA, free fatty acid; FAMEs, fatty acid methyl esters. *SAM2* encodes S-andenosyl-l-methionine synthetase; *FAA1* and *FAA4* encode fatty acyl-CoA synthetase; *ADO1* encodes adenosine kinase; *POX1* encodes acyl-CoA oxidase; *DmJHAMT* encodes O-Methyltransferase

Given that the FAEE and FAME yields achieved to date are well below commercial levels, additional efforts are required, particularly with respect to precursor supplies and enzyme activity. SAM and free fatty acids serve as FAME precursors, and efforts to increase their concentrations are thus needed to ensure efficient FAME biosynthesis. Many strategies for increasing free fatty acid concentrations in yeast cells have been explored to date. Zhou et al., for example, blocked fatty acid activation and degradation to generate yeast with high free fatty acid yields via the introduction of an optimized acetyl-CoA pathway, the expression of a fatty acid synthase with greater efficiency, and the overexpression of endogenous acetyl-CoA carboxylase (Zhou et al. 2016). In a fed-batch fermentation system, their engineered strain was able to achieve a free fatty acid yield as high as 10.4 g/L (Zhou et al. 2016). Methionine adenosyltransferase synthesizes SAM from ATP and methionine, and SAM subsequently serves as the primary biological methyl donor as it contains an active methylthioether group. To date, studies of yeast have revealed many genes involved in SAM accumulation, and metabolic engineering approaches can improve the efficiency of SAM synthesis in *S. cerevisiae* (Kanai et al. 2017). Zhao et al. established a yeast strain capable of more efficient SAM synthesis by combining *SAM2* gene overexpression and blocking the SAM decarboxylation pathway, achieving a SAM yield of 12.47 g/L with a fed-batch cultivation approach (Zhao et al. 2016). In this study, the deletions of the *FAA1, FAA2*, and *POX1* genes were conducted to enhance free fatty acid concentrations within *S. cerevisiae* cells, while *ADO1* deletion and *SAM2* overexpression were performed to enhance SAM accumulation (Fig. 4). The introduction of the *DmJHAMT* gene ultimately enabled a FAME yield of 5.79 ± 0.56 mg/L from these engineered yeast. This study represents the first report of the de novo synthesis of FAMEs in *S. cerevisiae.* While free fatty acid and SAM concentrations in these yeast cells were enhanced via metabolic engineering, this strategy only resulted in a FAME yield of 5.79 ± 0.56 mg/L. The largest barrier to the optimization of FAME production may be related to the suboptimal performance of the *Dm*JHAMT enzyme. Further protein engineering strategies focused on *Dm*JHAMT may represent a feasible means of enhancing FAME levels. Therefore, a series of rational design efforts based on known or simulated protein structures and directed evolution will be carried out in the future.

### Electronic supplementary material

Below is the link to the electronic supplementary material.

Supplementary Material 1

## Data Availability

All data supporting the findings of this study are available within the paper and its Supplementary Information.
