# Supplementary material for: Saccharomyces cerevisiae cellular engineering for the production of FAME biodiesel
Source: AMB Express. 2024 Apr 24;14:42. doi: 10.1186/s13568-024-01702-7 (PMC11043267; doi:10.1186/s13568-024-01702-7)
Supplement: Supplementary file 1 — Supplementary Material 1 [file 13568_2024_1702_MOESM1_ESM.pdf]

AMB Express

Saccharomyces cerevisiae cellular engineering for the production of FAME biodiesel

Laiyou Wang[1#], Bingbing Liu[1#], Qingshan Meng[2], Chunchun Yang[1], Yiyi Hu[1], Chunyan Wang[1], Pengyu Wu[1], Chen Ruan[1], Wenhuan Li[1], Shuang Cheng[1]* & Shuxian Guo[1]*

1, Henan Key Laboratory of Industrial Microbial Resources and Fermentation Technology, Nanyang Institute of Technology, Nanyang 473004, China

2, State Key Laboratory of Microbial Metabolism, Joint International Research Laboratory of Metabolic and Developmental Sciences, School of Life Sciences and Biotechnology, Shanghai Jiao Tong University, Shanghai, China

[#]These authors contributed equally to this work.

***Corresponding author:**

Shuang Cheng & Shuxian Guo

Henan Key Laboratory of Industrial Microbial Resources and Fermentation Technology, Nanyang Institute of Technology, Nanyang 473004, China

Email: crisycheng@163.com; guoshux@163.com

Supporting Information

Caption: Table S1. Strains and plasmids used in this study; **Table S2**. Primers used in this study; The nucleotide sequence of the codon-optimized *DmJHAMT* gene; **Figure S1** Schematic process of genome editing; **Figure S2** PCR identifications for the deletion of *FAA1*, *FFA4* and *POX1* genes; **Figure S3** Standard curves for the five kinds of FAMEs; **Figure S4** PCR identifications for the integration of *SAM2* and deletion of *ADO1*; **Figure S5** HPLC analysis of SAM; **Figure S6** Standard curve for SAM.

Table S1. Strains and plasmids used in this study

Strain or Plasmid	Characteristics	Reference/ Source
**Strains**		
Escherichia coli DH5α	F-φ80dlacZΔM15Δ(lacZYA-argF)U169 recA1 endA1 hsdR17(rK−, mK+) phoA supE44λ−thi-1 gyrA96 relA1	purchased from Beijing Catascis Biotech，Co.Ltd
Saccharomyces cerevisiae		
YPH499 (ATCC # 204679)	*MATa*, *ura3-52*, *lys2-801*, *ade2-101*, *trp1-D63*, *his3-D200*, *leu2-D1*	A gift from Tiangang Liu
SC01	YPH499 Δ*FAA1*	This study
SC02	YPH499 Δ*FAA1* Δ*FAA4*	This study
SC03	YPH499 Δ*FAA1* Δ*FAA4* Δ*POX1*	This study
SC04	YPH499 Δ*FAA1* Δ*FAA4* Δ*POX 1* XII-2::(PGK1p-*SAM2*)	This study
SC05	YPH499 Δ*FAA1* Δ*FAA4* Δ*POX1* XII-2::(PGK1p-*SAM2*) Δ*ADO1*	This study
SC06	SC05/pESC-HIS-1	This study
SC07	YPH499/pESC-HIS-1	This study
**Plasmid**		
pCRCT	Amp^R, *TEF1p-Cas9*, Containing *URA3* marker	(Addgene plasmid # 60621)
pDB78	Amp^R, Containing *HIS1* marker	Laboratory plasmid
pDB78-1	pDB78 derivative, *FAA1*gRNA	This study
pDB78-2	pDB78 derivative, *FAA4*gRNA	This study
pDB78-3	pDB78 derivative, *POX1*gRNA	This study
pDB78-4	pDB78 derivative, *SAM2*gRNA	This study
pDB78-5	pDB78 derivative, *ADO1*gRNA	This study

pESC-HIS	Amp^R^, Containing *HIS1* marker	Shanghai Zeye Biotechnology Co., Ltd
pESC-HIS-1	pESC-HIS derivative, *TPI1p-DmJHAMT*	This study

Table S2. Primers used in this study

Primers	Sequence (5'-3')	Description/Source
	Primers for construction of pDB78-1	
P1	TTGGAGCTCCACCGCGGTGGCGGCCGCTCTTTGAAAAGATAATGTATG	$P_{ScSNR52}$ forward
P2	GCTCTAAAACCTCGTCAGACCGCCTAACACGATCATTTATCTTTCACTGC	$P_{ScSNR52}$ reverse
P3	ATAAATGATCGTGTTAGGCGGTCTGACGAGGTTTTAGAGCTAGAAATAGC	crRNA forward
P4	GATAAGCTTGATATCGAATTCAGACATAAAAAACAAAAAAAGCACCACCGACTCGGTGC	crRNA reverse
	Primers for amplifying editing templates to delete gene *FAA1*	
P5	ACTTAGAATATGGATGATGCAG	*FAA1* upstream forward
P6	GGAAATGTTGATCCAATTGTTGTCTTTTTTTGTCT	*FAA1* upstream reverse
P7	AAAGACAACAATTGGATCAACATTTCCATGATAG	*FAA1* downstream forward
P8	TTGCTATGGTTTGTCTTCCATG	*FAA1* downstream reverse
	Primers for construction of pDB78-2	
P9	TTGGAGCTCCACCGCGGTGGCGGCCGCTCTTTGAAAAGATAATGTATG	$P_{ScSNR52}$ forward
P10	GCTCTAAAACCGTCCAATGTCGTGCATTACGATCATTTATCTTTCACTGC	$P_{ScSNR52}$ reverse
P11	ATAAATGATCGTAATGCACGACATTGGACGGTTTTAGAGCTAGAAATAGC	crRNA forward
P12	GATAAGCTTGATATCGAATTCAGACATAAAAAACAAAAAAAGCACCACCGACTCGGTGC	crRNA reverse
	Primers for amplifying editing templates to delete gene *FAA4*	
P13	CGGCTTTTTGGCTGCGCGTCTTTG	*FAA4* upstream forward
P14	AACTATGTCTTCCTTTTGATGCGTACTTCTTAG	*FAA4* upstream reverse
P15	GAAGTACGCATCAAAAGGAAGACATAGTTTTTTAC	*FAA4* downstream forward
P16	TTCAAACTTGGTGTACTATAG	*FAA4* downstream reverse
	Primers for construction of pDB78-3	

P17	TTGGAGCTCCACCGCGGTGGCGGCCGCTCTTGAAAAGATAATGTATG	$P_{ScSNR52}$ forward
P18	GCTCTAAAACTGTCCTAACTCAGTCATTGCGATCATTTATCTTTCACTGC	$P_{ScSNR52}$ reverse
P19	ATAAATGATCGCAATGACTGAGTTAGGACAGTTTTAGAGCTAGAAATAGC	crRNA forward
P20	GATAAGCTTGATATCGAATTCAGACATAAAAAACAAAAAAAGCACCACCGACTCGGTGC	crRNA reverse
	Primers for amplifying editing templates to delete gene *POX1*	
P21	CTTTTCTTAATTCTCTTTG	*POX1* upstream forward
P22	GAAACCTCTACATCGCAATACTAATTTATTATA	*POX1* upstream reverse
P23	GTATTGCGATGTAGAGGTTTCCTGTTTTCC	*POX1* downstream forward
P24	GATTGTTACCATAGCAACTCATG	*POX1* downstream reverse
	Primers for construction of pDB78-4	
P25	TTGGAGCTCCACCGCGGTGGCGGCCGCTCTTGAAAAGATAATGTATG	$P_{ScSNR52}$ forward
P26	GCTCTAAAACTGTAACGCGTTATGAAACTCGATCATTTATCTTTCACTGC	$P_{ScSNR52}$ reverse
P27	ATAAATGATCGAGTTTCATAACGCGTTACAGTTTTAGAGCTAGAAATAGC	crRNA forward
P28	GATAAGCTTGATATCGAATTCAGACATAAAAAACAAAAAAAGCACCACCGACTCGGTGC	crRNA reverse
	Primers for amplifying editing templates to integrate gene *SAM2*	
P29	GAGCGAACGTAAGAGAGGTTA	XII-2 upstream forward
P30	GATAATAGTATGAGGCGAATTTTCGCGTTTTGATG	XII-2 upstream reverse
P31	AAACGCGAAAATTCGCCTCATACTATTATCAGGGC	PGK1 promoter forward
P32	GTTTTGCTCTTGGACATTGTTTTATATTTGTTGTAAAAAG	PGK1 promoter reverse
P33	AACAAATATAAAACAATGTCCAAGAGCAAAACTTTC	*SAM2* gene forward
P34	TTTGAAAGATGATACTCTTTATTTCTAGACAGTTATATATTAAAATTCCAATTTCTTTG	*SAM2* gene reverse
P35	GTCTAGAAATAAAGAGTATCATCTTTCAAACGTTAATATTTCTGCTTTTTC	XII-2 downstream forward
P36	AATCCCATATGTGACGCAGCG	XII-2 downstream reverse
	Primers for construction of pDB78-5	

P37	TTGGAGCTCCACCGCGGTGGCGGCCGCTCTTTGAAA AGATAATGTATG	$P_{ScSNR52}$ forward
P38	GCTCTAAAACTCGGTAAGGACAAGTTCAGCGATCA TTTATCTTTCACTGC	$P_{ScSNR52}$ reverse
P39	ATAAATGATCGCTGAACTTGTCCTTACCGAGTTTTA GAGCTAGAAATAGC	crRNA forward
P40	GATAAGCTTGATATCGAATTCAGACATAAAAAACA AAAAAAGCACCACCGACTCGGTGC	crRNA reverse
	Primers for amplifying editing templates to delete gene *ADO1*	
P41	ACAGCTAAACATTTGCCCAAAAC	*ADO1* upstream forward
P42	GTAAGAAGAATAATTGCTTGCTCTTTCTTTTGC	*ADO1* upstream reverse
P43	AGCAAGCAATTATTCTTCTTACAATATAATAG	*ADO1* downstream forward
P44	GAGTTACACCAAGATAGTAGAAC	*ADO1* downstream reverse
	Primers for construction of pESC-HIS-1	
P45	GCCCTATAGTGAGTCGTATTACGGATCCCCTTCGAG ATTATATCTAGG	TPI1 promoter forward
P46	GCTTGGTTCATTTTTAGTTTATGTATGTG	TPI1 promoter reverse
P47	ATAAACTAAAAATGAACCAAGCTTCTCTTTACC	*DmJHAMT* gene forward
P48	CGGCCGCCCTTTAGTGAGGGTTGAATTC<u>TTTGAAAG ATGATACTCTTTATTTCTAGACAGTTATATA</u>TCAGTT GACACCTTTGACAA	*DmJHAMT* gene reverse
	Primers used to identify the deletion of gene *FAA1*	
P49	ACTTAGAATATGGATGATGCAG	forward
P50	TGAATTAGCCCTTTCTCTCCC	reverse
	Primers used to identify the deletion of gene *FAA4*	
P51	GACAAAAGCGCAAACCGAACCG	forward
P52	AATTTGGACAACAGCTGGTTG	reverse
	Primers used to identify the deletion of gene *POX1*	
P53	CTGAACAACTAATCAAAATATC	forward
P54	ACGCGCGTACCCAATTGAGGATC	reverse
	Primers used to identify the integration of gene *SAM2*	
P55	CTATCAGTCCAATGACAGTA	forward
P56	TAATTACTTCCTTGATGATCT	reverse
	Primers used to identify the deletion of gene *ADO1*	
P57	GGGATGTCCGCTTACTAATTCC	forward
P58	GGTTTGCTGTTCTCTTTAGCA	reverse

The underlined sequence is a reverse complementary sequence of the synthesized Tguo1 terminator.

The nucleotide sequence of the codon-optimized *DmJHAMT* gene

ATGAACCAAGCTTCTCTTTACCAACACGCTAACCAAGTCCAAAGACACGATGCCAAGT
TGATTTTGGACGAATTTGCTTCTACTTTACAATGGAGATCTGATGGTGAAGATGCTTTGT
TGGATGTCGGTTCCGGCTCTGGTAACGTTTTGATGGACTTTGTCAAGCCTTTGTTACCA
TCTAGAGGTCAATTGGTCGGTACTGACATTTCCAGTCAAATGGTTGGTTACGCTTCCAA
GCACTACCAACGTGAAGAACGTACCAGATTCCAAGTTTTGGACATCGGTTGTGAAAGA
TTGCCACAAGAATTATCTGGTAGATTCGACCACGTTACCTCTTTCTACTGCTTGCATTGG
GTTCAAAACTTGAAGGGTGCTTTGGGTAACATCTATAACTTGTTGAGACCAGAGGGTG
GTGACTGTTTGTTGGCTTTCCTAGCCTCCAACCCAGTTTACGAAGTCTATAAAATCTTA
AAGACTAACGAAAAGTGGTCTTCTTACATGCAAGATGTTGAACAATTCATTTCACCTCT
ACATTACTCCTTGAACCCAGGTGAAGAATTCTCTCAACTGTTGAATGAAGTTGGTTTCA
TTCACCACAACGTTGAAATCAGAAACGAAGTTTTCGTCTACGAAGGTGTTAGAACTTT
GAAGGACAACGTCAAGGCTATCTGTCCATTCTTGGAAAGAATGCCAGCTACCTTGCAC
GAAGATTTCTTAGATGACTTCATCGAAATTGTTATCTCCATGAACTTGCAACAGGGTGA
AAACAACGAAGACCAAAAGTTTTTGAGCCCATACAAGTTGGTCGTTGCTTACGCCAGA
AAGTCTCCAGAATTCGTTAATAATGTGTTGTTGGAACCACCACACCAATCCATTGTCAA
AGGTGTCAACTGA

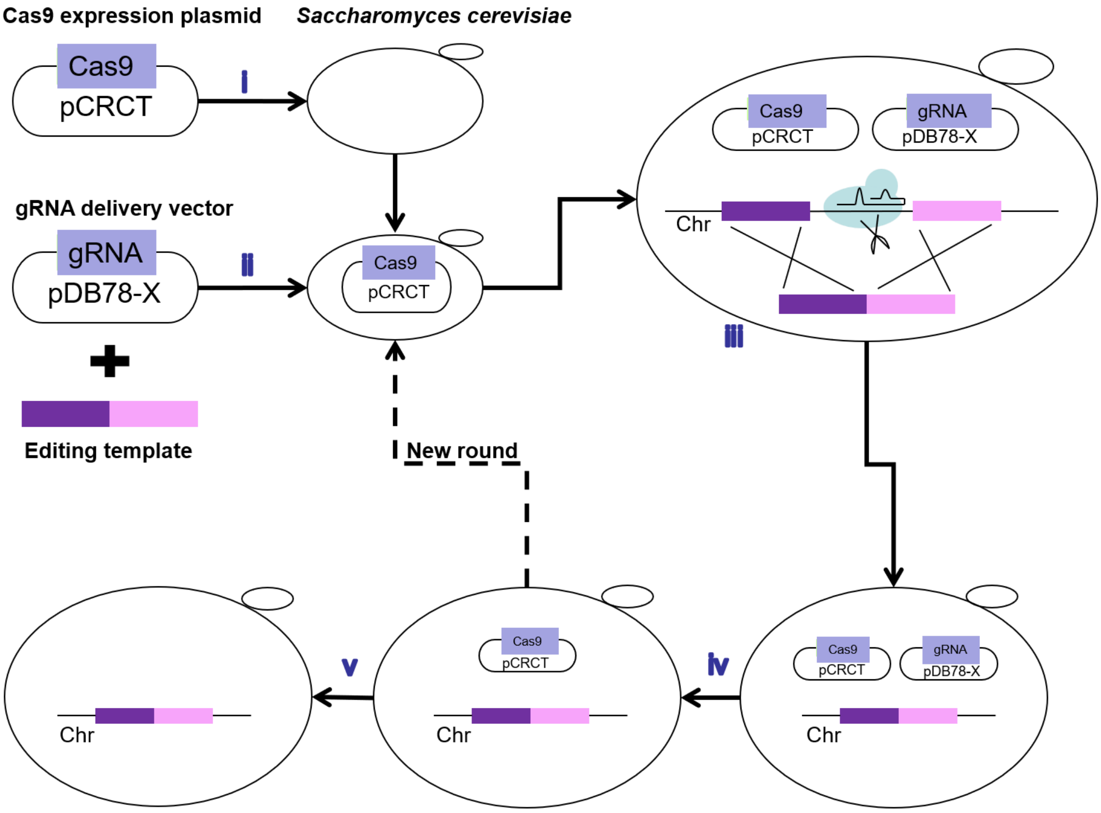

Figure S1 Schematic process of genome editing. (i) Transformation of the Cas9 expression plasmid pCRCT. (ii) Transformation of the gRNA delivery vector and editing template. (iii) Genome editing. (iv) Elimination of the gRNA delivery vector. (v) Elimination of the Cas9 expression plasmid. Chr: chromosomal DNA.

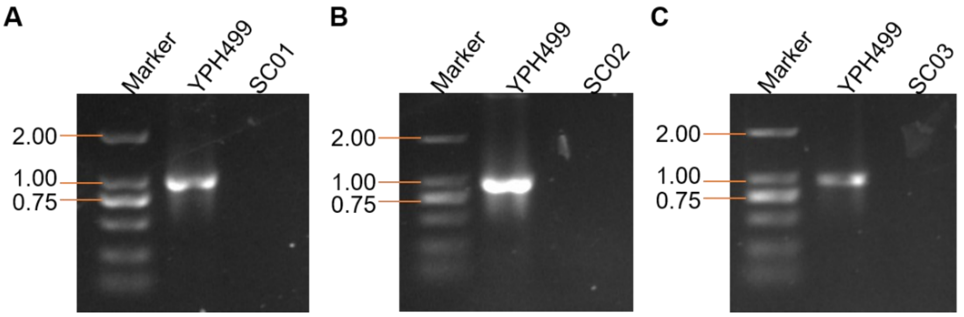

Figure S2 PCR identifications for the deletion of *FAA1* (A), *FFA4* (B) and *POX1* (C) genes using primers within and without the gene deleted, respectively. A: The strain YPH499 yielded a 1000-bp fragment, while the Δ*FAA1* mutant yielded no fragment. B: The strain YPH499 yielded a 995-bp fragment, while the Δ*FAA4* mutant yielded no fragment. C: The strain YPH499 yielded a 999-bp fragment, while the Δ*POX1* mutant yielded no fragment.

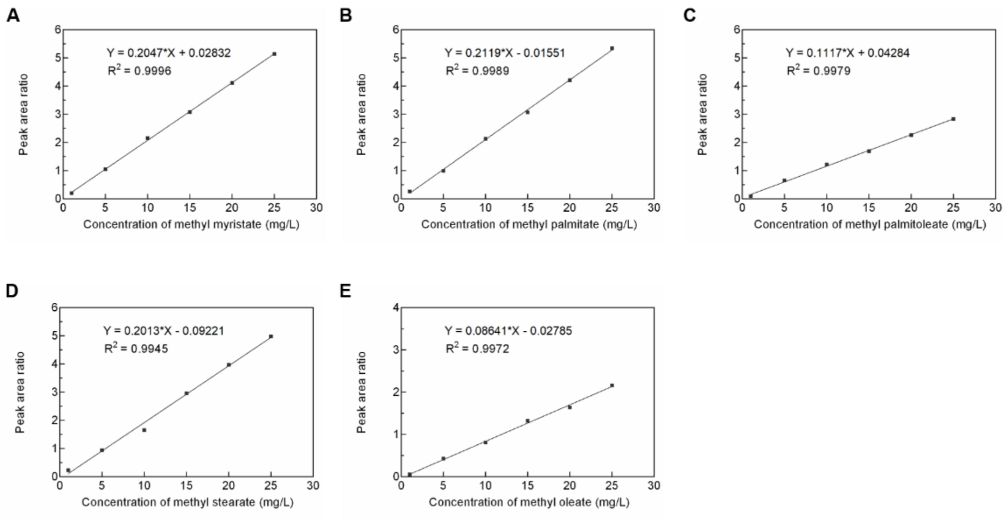

Figure S3 Standard curves for the five kinds of FAMEs [C14:0 (A), C16:0 (B), C16:1 (C), C18:0 (D) and C18:1 (E)].

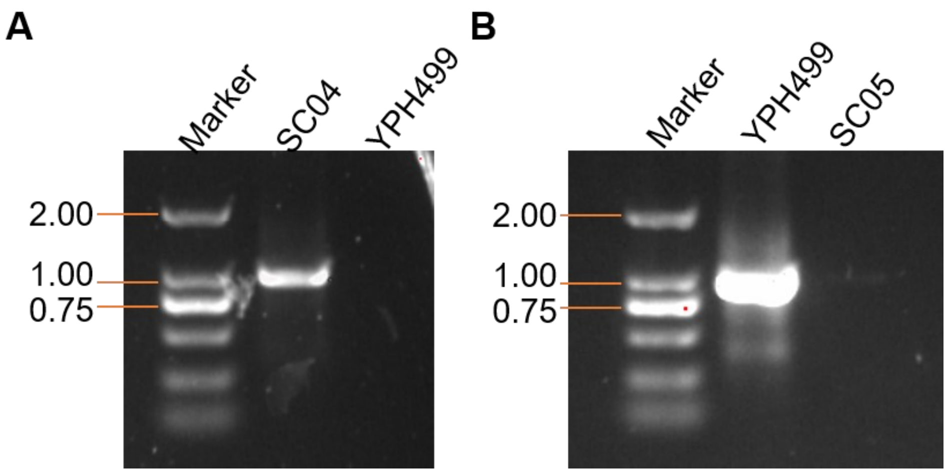

Figure S4 PCR identifications for the integration of *SAM2* (A) and deletion of *ADO1* (B) using primers within and without the target gene, respectively. A: The strain SC04 yielded a 1007-bp fragment, while the strain YPH499 yielded no fragment. B: The strain YPH499 yielded a 995-bp fragment, while the Δ*ADO1* mutant yielded no fragment.

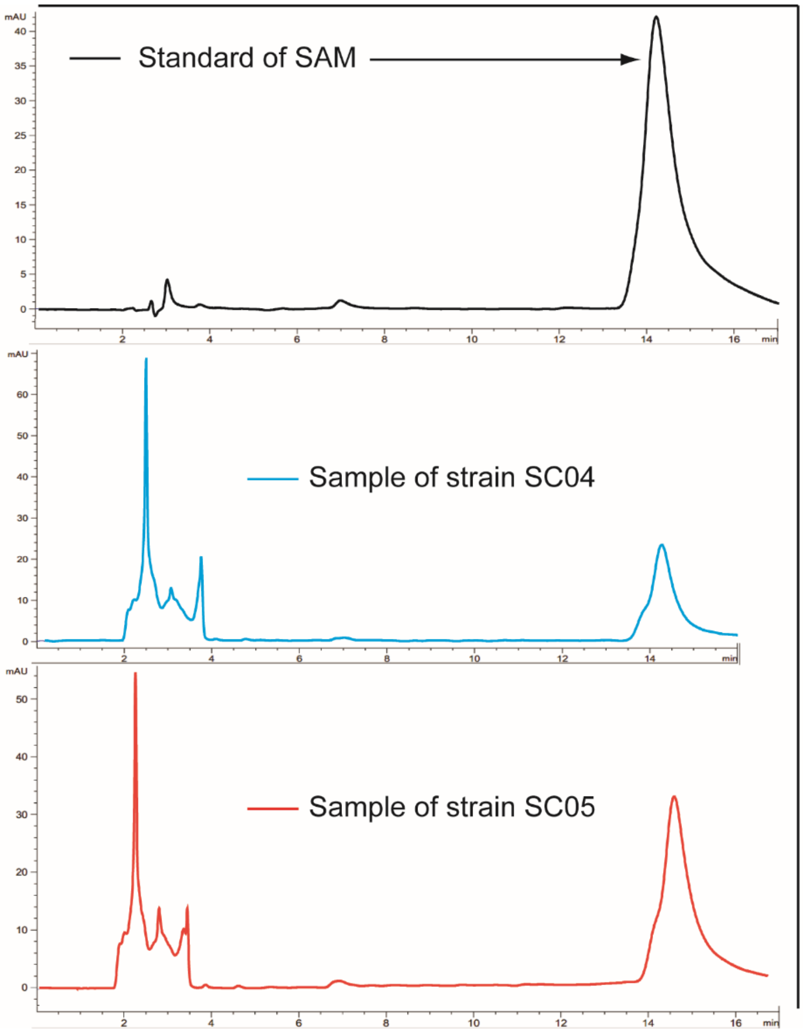

Figure S5 HPLC analysis of SAM.

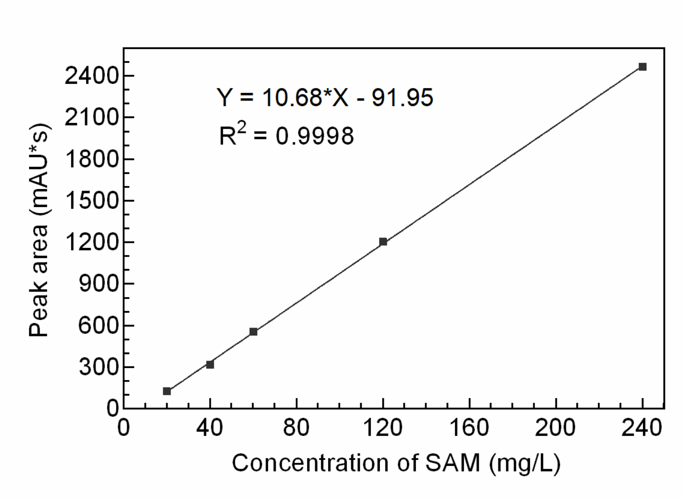

Figure S6 Standard curve for SAM.
